# Correction

**DOI:** 10.1080/02813432.2026.2667708

**Published:** 2026-05-15

**Article title:** Persistent physical symptoms not explained by structural abnormalities or disease processes: a primary care approach to promote recovery

**Authors:** Abrahamsen, C., Beadsworth, M., Bostock, W., Chalder, T., Flottorp, S., Fors, E. A., Garner, P., Hadfield, S., Kennedy, B., Kuehn, R., Landmark, L., Launes, G., Liira, H., Linnestad, L., Rotkirch Virrantaus, H., and Vangelova-Korpinen, V.

**Journal:** *Scandinavian Journal of Primary Health Care*

**Bibliometrics:** Volume 44, Number 1, pages 2633765

**DOI:** https://doi.org/10.1080/02813432.2026.2633765

When this article was first published the Disclosure Statement was omitted this has now been included in the article as follows:

**Disclosure statement**

All authors are members of The Oslo Chronic Fatigue Network, a group that all have a declared **biopsychosocial understanding** of long-term fatigue and related persistent physical symptoms.

**Cathrine Abrahamsen**

Cathrine Abrahamsen developed the structured communication tool (ICIT), which was evaluated in an RCT, showing positive effects. She taught use of the tool to general practitioners (GPs) and received fees for this training. There are no financial royalties associated with the use of the ICIT.

**Mike Beadsworth**

None declared.

**Will Bostock**

Will Bostock is the director of Cambridge Progressive Medicine Ltd, providing GP services to the NHS.

**Trudie Chalder**

Trudie Chalder has written self-help books on chronic fatigue and has received ad hoc payments for workshops on long-term conditions, as well as travel expenses and accommodation costs for attending conferences. She was on the Expert Advisory Panel for the Covid-19 Rapid Guidelines and is in receipt of research grants from Guy’s and St Thomas’ Charity, the NIHR, and UKRI. She is the Director of the Persistent Physical Symptom service at South London and Maudsley NHS Trust.

**Signe Flottorp**

None declared.

**Egil A Fors**

Member of the Medical Advisory Board for the Norwegian Fibromyalgia Association; this is an unpaid position.

**Paul Garner**

In receipt of travel expenses from the Long Covid EU Horizon Project. Member of the Consumer Advisory Group for the Collaborative on Fatigue and associated symptoms Following Infection.

**Sarah Hadfield**

Sarah Hadfield has a small private practice working with patients using mind-body medicine techniques. She also sits on the SIRPA (Stress Illness Recovery Practitioner Association) advisory board and serves as a medical advisor for Living Proof, a not-for-profit organisation promoting the mind-body approach.

**Rebecca Kennedy**

Owns a private practice where she treats patients with long COVID and CFS/ME using a mind-body approach. Board Member of the Association for Treatment of Neuroplastic Symptoms.

**Rebecca Kuehn**

None declared.

**Live Landmark**

Live Landmark receives honoraria for lectures on stress and coping and is a Lightning Process practitioner.

**Gunvor Launes**

In receipt of honoraria for public lectures on mental health, life stressors and exhaustion in public health.

**Helena Liira**

Helena Liira is the former Editor in Chief of the Scandinavian Journal of Primary Health Care, and co-ordinator of the Long Covid EU Horizon Project.

**Lina Linnestad**

No conflicts of interest

**Hélène Rotkirch Virrantaus**

In receipt of research grants from the Long Covid EU Horizon Project and the Gyllenberg Foundation in Finland. She has additionally received lecturing fees from the City of Helsinki, the pharmaceutical company Teva Finland, the Junior Doctors’ Association in Finland, the Association for General Practitioners in Finland, and the Finnish Medical Association.

**Velina Vangelova-Korpinen**

In receipt of research grants from the Long Covid EU Horizon Project and the Gyllenberg foundation in Finland, lecturing fees from the City of Helsinki, Terveystalo, The Association for General Practitioners in Finland, and The Finnish Medical Association.

A number of minor corrections have also been updated on the article.

